# Mammal community composition and season determine the abundance of questing ticks in mountainous forests of central Japan

**DOI:** 10.1016/j.ijppaw.2025.101120

**Published:** 2025-07-21

**Authors:** Hayato Iijima, Kaori Morishima, Hirotaka Komine, Yuya Watari, Kandai Doi, Kimiko Okabe

**Affiliations:** a Forestry and Forest Products Research Institute, Matsunosato 1, Tsukuba, Ibaraki, 305-8687, Japan; b Faculty of Agriculture, Yamagata University, 1-23, Wakaba-machi, Tsuruoka, Yamagata, 997-0037, Japan

**Keywords:** *Haemaphysalis*, Severe fever with thrombocytopenia syndrome, Sika deer, Tick-borne diseases, Tick phenology

## Abstract

Understanding the ecological drivers of tick abundance is crucial to mitigate the growing risk of tick-borne diseases such as severe fever with thrombocytopenia syndrome (SFTS) in Japan. This study investigates how mammal community composition and seasonality affect the abundance of questing ticks in mountainous forests of Gifu Prefecture, a border region of the SFTS endemic zone. Camera traps were used to monitor mammal species, and questing ticks were collected monthly via flagging along transects in 10 forest plots during 2021–2022. We recorded 14 mammal species including small-sized mammals like mice, medium-sized mammals like raccoon dog (*Nyctereutes viverrinus*), and large-sized mammals like wild boar (*Sus scrofa*). Among them, sika deer (*Cervus nippon*) was the most abundant. A total of 408 adult, 292 nymphal, and 1480 larval ticks representing 11 species (five *Haemaphysalis* species, five *Ixodes* species, and *Amblyomma testudinarium*) were collected. Generalized linear mixed models revealed that the abundance of adult *Haemaphysalis kitaokai* and *H. megaspinosa*, and nymphal *H. flava* and *H. megaspinosa* were significantly associated with sika deer abundance. Seasonal patterns varied by species and life stage, with *H. kitaokai* adults peaking in spring and early winter, and *H. megaspinosa* adults and nymphs in autumn. Wild boar abundance did not significantly influence tick numbers. These findings highlight the importance of sika deer as a key host driving tick population dynamics and underscore the role of host-targeted management, particularly deer population control, as a potential strategy to reduce tick density and related disease risks. Long-term monitoring is essential given ongoing climate and land-use changes that may alter tick phenology and distribution. Our results contribute to region-specific understanding of tick ecology and support the development of effective, ecologically informed countermeasures against tick-borne diseases in Japan.

## Highlights

• Sika deer abundance was associated with higher questing tick abundance.
• Tick abundance was generally high in spring and autumn but differed among tick species.
• Deer population control is suggested as an ecological strategy to reduce tick abundance.

## Graphical abstract

**Figure:**
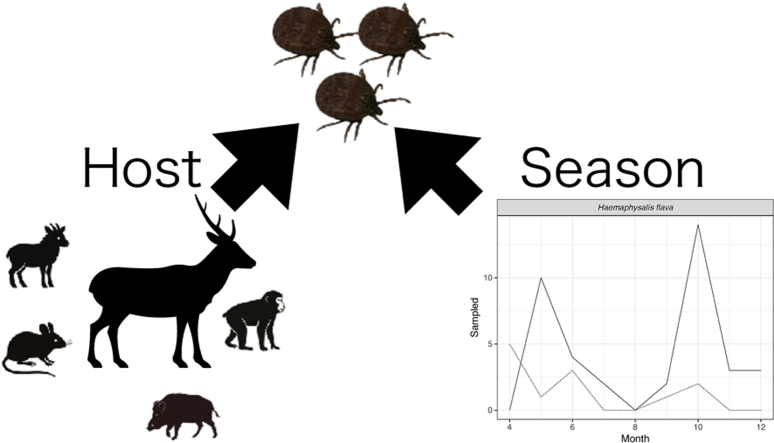

## 1 Introduction

Vector-borne diseases, many of which originate from wildlife, are a growing concern in relation to biodiversity loss and climate change (Civitello et al., 2015; Githeko et al., 2000; Ostfeld and Keesing, 2000; Taylor et al., 2001). In the temperate zone, ticks are the primary vectors of zoonoses in North America, Europe, and Asia (Lane, 2009; Rochlin and Toledo, 2020). Ticks that transmit pathogens suck blood only once at each developmental stage (larva, nymph, or adult), and consequently utilize three different hosts during a lifetime (Keirans, 2009; Madison-Antenucci et al., 2020; Oliver Jr, 1989). Recently, changes in distribution and population densities of wildlife and vector ticks are expected under accelerating climate change, ecosystem degradation (Dantas-Torres, 2015; Ostfeld and Keesing, 2000; Pfäffle et al., 2013), and the reduction of hunting activity (Manfredo et al., 2017). Then, immediate preventive measure for TBDs need to be developed, especially at the border areas of infection (Eisen and Eisen, 2018; Gray et al., 2009).

Among Asian TBDs, severe fever with thrombocytopenia syndrome (SFTS), caused by the SFTS virus (SFTSV) is a new one, first identified in western Japan in 2013 following its initial description in China in 2011 (Takahashi et al., 2014; Yu et al., 2011). It has since spread within China, Japan, South Korea, Taiwan, Myanmar, and Vietnam (Iijima et al., 2025; Kobayashi et al., 2020; Lin et al., 2020; Park et al., 2021; Win et al., 2020). SFTS is a major concern among TBDs in Japan, as it continues to expand from western to eastern Japan each year, with high mortality as high as 27 % in Japan (Kobayashi et al., 2020). Although the mortality rate by SFTS is particularly high among individuals over 60 years, effective treatments for this viral disease remain very limited (Yamaji et al., 2018; Yokomizo et al., 2022). In Asia, *Haemaphysalis* *logicornis* *and H. flava* are the two suspected vectors that transmitting SFTSV (Casel et al., 2021; Fang et al., 2023) but key pathogen reservoir and tick-host animals are different, with livestock in mainland Asia and wildlife in Japan (Wang et al., 2021). More than 80 % of SFTS cases are farmers in China (Sun et al., 2017), but no such information is available in Japan.

Among wildlife,the expansion and increase of large mammals, such as the sika deer (*Cervus nippon*; Iijima et al., 2023); and wild boar (*Sus scrofa*), are suspected to affect tick populations especially in Japanese forest areas (Iijima et al., 2022; Matsuyama et al., 2022; Okabe et al., 2019; Tabara et al., 2019). In Tochigi Prefecture, located in eastern part of Japan, the numbers of questing adult *H. flava* and *H. kitaokai*, as well as nymphal *H. flava*, *H. longicornis*, and *H. megaspinosa*, were high in areas where the number of sika deer photo captured by camera traps was also high (Iijima et al., 2022). However, because mammal community composition (i.e., species richness and relative abundances) varies across regions, the relationship between questing tick abundance and hosts should be investigated in different areas.

The phenology (i.e., seasonal fluctuation of the number of questing ticks) of ticks is also important information for reducing the risk of tick bite. In Gifu Prefecture, central Japan, previous surveys reported the collection of *H*. *flava*, *H*. *kitaokai*, and *H*. *longicornis* in May, October, and November by flagging method (Kobayashi et al., 2021; Maeda and Kadosaka, 2016) and *A*. *testudinarium*, *H*. *flava*, *H*. *japonica*, *H*. *longicornis*, *H*. *megaspinosa*, *I*. *monospinosus*, *I. nipponensis*, *I*. *ovatus*, and *I*. *persulcatus* collected from animal hosts, including humans (Kubo et al., 2008; Maeda and Kadosaka, 2016; Sanda et al., 1987; Shimada et al., 2003). However, these previous studies were conducted only one time or during one year. To clarify the phenology of questing ticks, seasonal surveys should be conducted over at least two years to account for annual fluctuations in tick abundance. Furthermore, because climate change may alter the phenology of questing ticks (Levi et al., 2015), the data of questing phenology is important but such data is still limited (Ostfeld and Brunner, 2015).

**Fig. 1:**
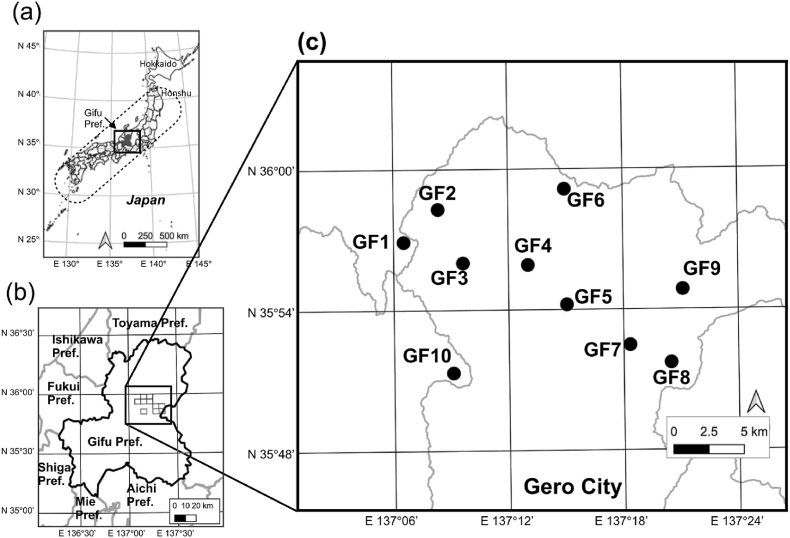
(a) Location of Gifu Prefecture (Pref.) in Japan. (b) Location of Gifu Prefecture and the surrounding area. (c) Locations of the 10 plots in Gero City.

Consequently, we set the objective of our study to clarify the effects of mammal community composition and season on the abundance of questing ticks, which may be infected with SFTSV. We assess the current status of ticks and wildlife in Gero City in Gifu Prefecture, Japan, which is the border area of SFTS endemic zone for further development of preventive strategy. Given that SFTSV was detected from ticks in adjacent Fukui Prefecture, the border of which locates approximately 20 km to the west of Gero City (Ishiguro et al., 2014), and that SFTS cases were reported in the surrounding administrative districts (i.e., Toyama, Ishikawa, Fukui, Shiga, Mie, and Aichi Prefectures; https://www.niid.go.jp/niid/ja/id/2245-disease-based/sa/sfts/idsc/idwr-sokuhou/7415-sfts-nesid.html) and Nakatsugawa City where locates approximately 50 km to the southeast of Gero City (Fig. 1), so preventive measures are urgently needed in the area.

## 2 Materials and methods

### 2.1 Study site

We established 10 plots (GF1 to GF10) in national forests, Gifu prefectural forests, and public forests in Gero City, Gifu Prefecture, Japan (Fig. 1). The elevation of each plot ranged from 587 to 935 m. In 2021, the mean temperature in Gero City (N 35° 53.3′, E 137° 12.4’) was 13.1 °C, with a minimum and maximum of −9.4 °C and 36.0 °C, respectively. The annual precipitation in Gero City in 2021 was 3191 mm. Meteorological data were obtained from https://www.data.jma.go.jp/obd/stats/etrn/. Approximately 90 % of the land of the city is covered by forest (https://www.city.gero.lg.jp/soshiki/15/1166.html, accessed December 20, 2024). Approximately 60 % of the forest consists of plantations of *Cryptomeria japonica* (L.f.) D.Don, *Chamaecyparis obtusa* (Siebold et Zucc.) Endl., *Pinus densiflora* Siebold et Zucc., and *Larix kaempferi* (Lamb.) Carrière (https://www.city.gero.lg.jp/soshiki/15/1166.html, accessed December 20, 2024).

### 2.2 Mammal survey

To analyze the relationship between tick and host animals, we deployed two sensor camera traps (LTL Acorn, Ltl-6210MC Plus 940NM, Cams) on tree trunks about 50 cm above the ground in each plot. To improve the accuracy of the identification of captured wildlife, two pictures were taken when the camera was triggered and there was a minimum of 10 min interval between two triggers. We checked the camera traps every 3–4 months and replaced the batteries and memory cards from April 2021 to December 2022. However, owing to deep snow, we were unable to conduct surveys from January to March. In the GF1 plot, as one camera was stolen, we only collected data from one sensor camera trap from April to June 2021, so we corrected the number of shots for the number of days for which the camera traps were operational in each trapping plot. We calculated the species abundance index in each plot by counting the number of shots for each species. Although animals were not generally identified at the individual level, when they were sitting in front of the camera continuously (>30 min), they were considered the same individual and counted the photos as one trigger. Furthermore, we confirmed the reliability of the number of shots as abundance index by comparing the number of sika deer shots with the estimates of sika deer abundance gradient among our plots by (Ando et al., 2023). The number of photographed sika deer and estimation by previous study significantly correlated (Pearson's product-moment correlation, r = 0.665, p = 0.036). Therefore, we used the number of shots as an index of abundance, although this assumption was only applicable to sika deer in the case described above.

### 2.3 Questing tick collection

From April to December in 2021 and 2022, respectively, we collected questing ticks in the plots every month. To collect ticks, we established a 100 m line transect at forest edge along a forest road in each plot and used a white flannel cloth (0.7 m in width and 1.2 m in length) to flag vegetation (shorter than 0.5 m in height) and the litter layer on the transect. We collected tick only at forest edge because the number of sampled ticks in forest edge were larger than those in forest interior or on forest load (Iijima et al., 2022). At every 10 m of flagging on the transect, we checked both sides of the cloth surface and collect adults and nymphs into a vial with wet tissue. Larval ticks that remained on the cloth until the end of the sampling period were collected in the laboratory, as sometimes hundreds of larvae aggregated in a spot on the cloth. Ticks were identified under a stereomicroscope (10–120 × magnification) in accordance with the morphological identification keys (Fujita and Takada, 2007; Yamaguti et al., 1971; Yamauchi and Takada, 2015). Because some tick species in immature stages are rarely sampled by flagging (e.g., larval and nymphal *Ixodes ovatus* (Takada et al., 2019), we conducted statistical analysis about nymph only for tick species that could be collected by flagging.

### 2.4 Data analyses

To determine the effects of mammal community and season on adult and nymphal tick abundance collected by the flagging method, we constructed a generalized linear mixed model (GLMM) with Poisson error structure and log link function. The response variable of GLMM was the number of sampled ticks by each plot, each month, and each tick species. The explanatory variables of GLMM were first (PC1) and second axis scores (PC2) of PCA about mammal community (i.e., the matrix of the total number of photos for each mammal species and each plot during the study period), mammal species richness (total number of mammal species present at a site), and each month. We used the scores for avoiding multicollinearity among the number of each mammal species photo. We calculated mammal species richness for each plot based on the photo data. Each plot and year were included as random effects. Because the numbers of sampled tick for some tick species or developmental stages are limited, we fitted GLMM only for adult *H. kitaokai*, adult *H. megaspinosa*, nymphal *H. flava*, and nymphal *H. megaspinosa*, separately. Because we analyzed the full model (i.e., all explanatory variables were included), we run four models. Since those species could infest on the same host species at all developmental stages in the same areas (Kitaoka et al., 1975; Mori et al., 1990; Yamaguti et al., 1971; Yamauchi, 2003), we assumed that the relationship between questing tick abundance and host abundance was static in a plot regardless of host migration and analyzed the relationship between the number of collected ticks at each month and the summed number of photographs of each mammal species during two years. We fitted GLMM by lme4 package (Bates et al., 2015) of R (R Core Team, 2024). We conducted Tukey's multiple comparison test among month by multcomp package (Hothorn et al., 2008) of R (R Core Team, 2024). We did not analyze the larval ticks with the GLMM because the sampling method differed from the adult and nymphal tick sampling method. The number of sample larval ticks were shown in Fig. S1.

## 3 Results

### 3.1 Sampled mammals and questing ticks

**Fig. 2:**
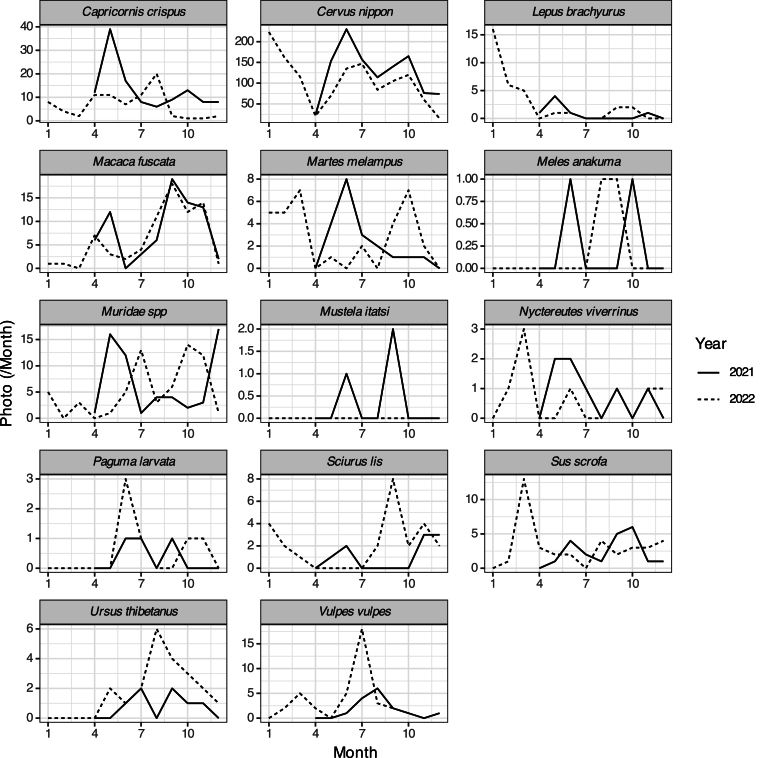
The number of monthly photographed mammals.

Among 14 mammal species recorded in all camera traps, sika deer were most frequently captured (Table 1). Japanese serow (*Capricornis crispus*), Japanese monkey (*Macaca uscata*), mice (*Muridae* spp.), Japanese marten (*Martes melampus*), and wild boar (*Sus scrofa*) were also abundant (Table 1). Although we did not obtain the camera data of winter in 2021, sika deer were relatively abundant in May, June, October, and January (Fig. 2; Table S1). Japanese monkey was relatively abundant in May, September, October, and November (Fig. 2; Table S1). The seasonal occurrence pattern of other mammals differed annually (Fig. 2; Table S1).

**Table 1:** The number of annually photographed mammals.

Species	Common name	2021	2022
*Capricornis crispus*	Japanese serow	120	80
*Cervus nippon*	Sika deer	1132	1258
*Lepus brachyurus*	Japanese hare	7	33
*Macaca fuscata*	Japanese macaque	75	74
*Martes melampus*	Japanese marten	20	33
*Meles anakuma*	Japanese badger	2	2
*Muridae* spp		60	63
*Mustela itatsi*	Japanese weasel	3	0
*Nyctereutes viverrinus*	Raccoon dog	7	8
*Paguma larvata*	Masked palm civet	3	6
*Sciurus lis*	Japanese squirrel	9	25
*Sus scrofa*	Wild boar	21	37
*Ursus thibetanus*	Asiatic black bear	7	21
*Vulpes vulpes*	Red fox	15	39

If individuals were sitting in front of the camera continuously (>30 min), they were considered the same individual and counted the photos as one trigger.

**Fig. 3:**
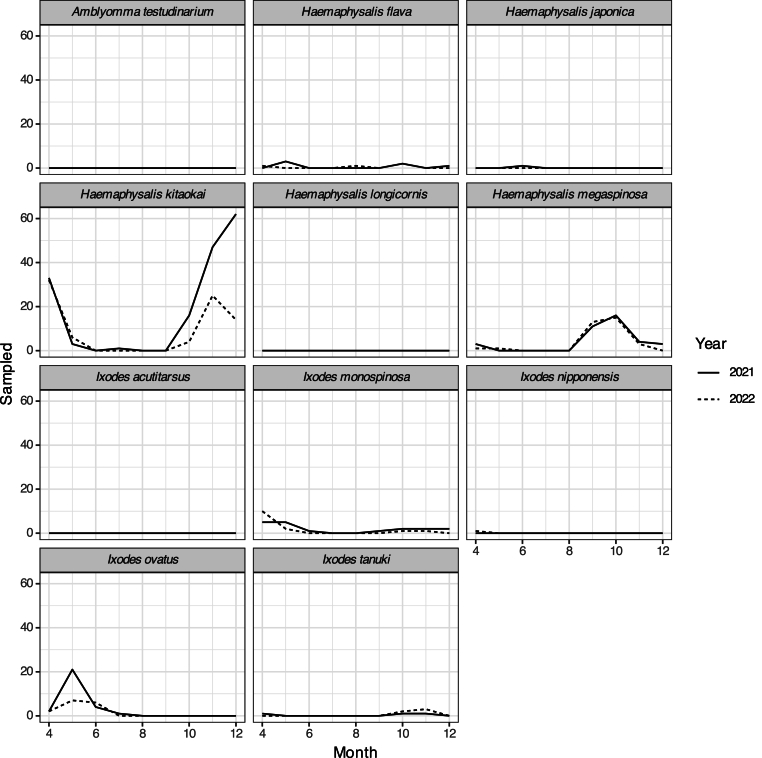
Seasonal difference of sampled adult ticks.

**Fig. 4:**
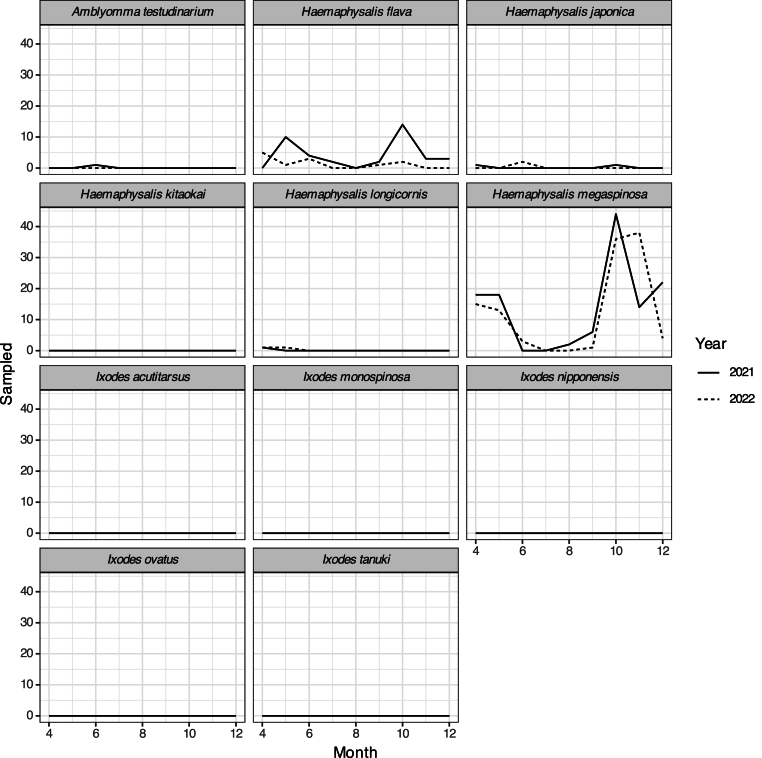
Seasonal difference of sampled nymphal ticks.

We collected 408 questing adult ticks, 292 nymphal ticks, and 1480 larval ticks during the two years. They belong to 11 species and three genera (Table S2). The phenology was consistent within each tick species over the two years (Fig. 3, Fig. 4). The dominant species were also consistent over the two years although with a little difference in collected individual numbers (Fig. 3, Fig. 4). Adult *H. kitaokai* were abundant in April (n = 33 and 32 for 2021 and 2022, respectively; Table S2), November (n = 47 and 25), and December (n = 62 and 14). Adult *H. megaspinosa* was abundant in September (n = 11 and 13) and October (n = 16 and 15). Nymphal *H. megaspinosa* was abundant in October (n = 44 and 36) and November (n = 14 and 38). Nymphal *H. flava* was abundant in May (n = 10 and 1) and October (n = 14 and 2).

### 3.2 Effects of mammal community on questing tick abundance

**Fig. 5:**
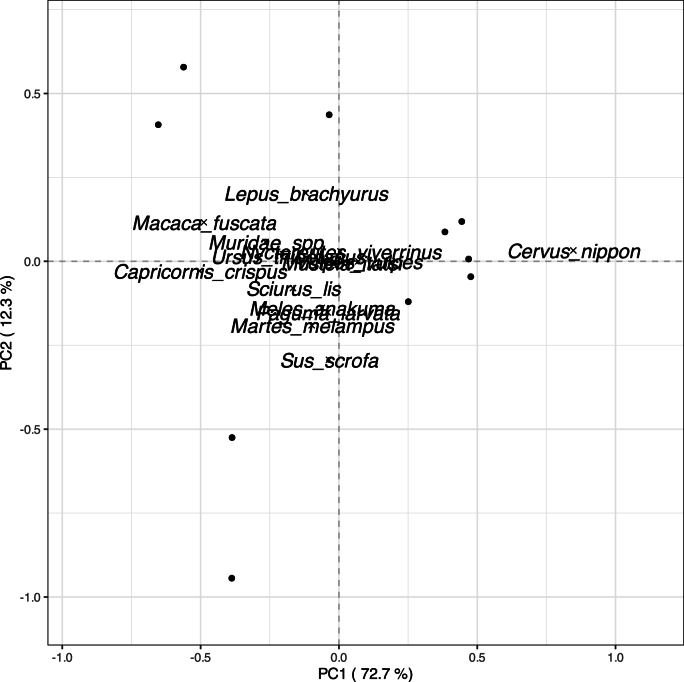
Biplot of PCA about mammal community The solid circle and cross symbol indicate the scores of site and host species, respectively.

PCA well explained mammal community because the percentages of explained variance by PC1 (72.7 %) and PC2 (12.3 %) were high (Fig. 5). PC1 was mainly related to the number of photographed sika deer (Fig. 5). PC2 was mainly related to the number of photographed wild boar (Fig. 5).

For all targeted tick species and developmental stages, PC1 significantly increased questing tick abundance, but PC2 did not affect it (Table 2). Mammal species richness affected tick abundance only for adult *H. megaspinosa* (Table 2). Adult *H. kitaokai* was significantly abundant in April, November, and December (Table 2). Adult *H. megaspinosa* was significantly abundant in September and October (Table 2). Abundances of nymphal *H. flava* did not differ seasonally (Table 2). Nymphal *H. megaspinosa* was significantly abundant in October and November (Table 2).

**Table 2:** Summary statistics of GLMM about the number of sampled ticks.

Explanatory	Adult				Nymph			
variables	*H. kitaokai*		*H. megaspinosa*		*H. flava*		*H. megaspinosa*	
Intercept	−0.589	a	−5.603	a	−3.230	a	−3.504	ab
Month5					0.788	a	−0.063	ab
Month6					0.336	a		
Month9			1.792	b				
Month10	−1.179	b	2.048	b	1.163	a	0.886	c
Month11	0.102	a	0.560	a			0.455	ac
Month12	0.156	a					−0.239	b
PC1	5.985	0.00	7.151	0.01	3.129	0.00	4.461	0.00
PC2	−1.963	n.s.	−2.683	n.s.	0.667	n.s.	−2.022	n.s.
Mammal species richness	−0.056	n.s.	0.203	0.05	0.122	n.s.	0.269	n.s.

*H.* is the abbreviation of *Haemaphysalis*. *P*-values were based on Wald's test. Intercept term is the reference category of month (i.e., April). Different letters among month indicates the statistical significant difference evaluated by Tukey's multiple comparison test (*p* < 0.05).

## 4 Discussion

We collected nine species of ticks belonging to three genera that have previously been recorded in Gifu Prefecture (Table S2), except for *I*. *nipponensis* and *I.* *persulcatus* which were not found at the survey sites in Gero city, likely due to differences in sampling locations. Ticks collected in this study were common species found in mainland Japan, except for Hokkaido, northern part of Japan. Among them, it should be noted that we collected a nymphal *A*. *testudinarium* because it was once considered as a southern species, primarily based on old sample localities in Southeast Asia and the western part of Japan (Yamaguti et al., 1971), but it now has been reported in Aomori Prefecture, the northeastern part of Honshu (Doi, 2023; Doi et al., 2021; Shimada et al., 2022; Takahashi et al., 2021; Terada et al., 2019; Yamaguti and Kitaoka, 1980). We suggest that *A. testudinarium* should be continuously monitored because it is responsible for many human tick-bite cases in Japan (Natsuaki, 2021; Shimada et al., 2022). In addition, to consider the ticks as the vectors of tick-borne diseases, *H. longicornis* and *H. flava* are suspected SFTS vectors among our samples (Casel et al., 2021).

We also found that the questing season of adult *H. kitaokai* and *H. megaspinosa,* the dominant tick species amongst our study sites (Fig. 3, Fig. 4, Table S2), was consistent with previous studies: adults of *H*. *kitaokai* were reported from October to June, and adults of *H*. *megaspinosa* were found from September to May, with a few exceptions in summer flagging collections in Japan (Iijima et al., 2022; Kitaoka et al., 1975; Nakajima et al., 1991; Shibata et al., 2020; Yamauchi et al., 2009). The phenology of most other tick species was consistent with previous studies conducted around the area or in northern Honshu. For instance, nymphal *A. testudinarium* appeared in late spring to early summer (Okumura et al., 1987), adult and nymphal *H*. *japonica* emerged in late spring to early summer and fall, respectively (Ishiguro et al., 1992, 2013), nymphal and larval *H*. *longicornis* emerged in late spring and fall, respectively (Ishiguro et al., 2014; Maeda and Kadosaka, 2016), larval *I. acutitarsus* emerged in fall (Takada et al., 1978), adult *I*. *monospinosus* could be found anytime except for summer (Ishiguro et al., 2008, 2013; Kitaoka et al., 1975), and adult *I. tanuki* emerged in fall (Fujita et al., 2013). However, this phenology of tick may change in near future. Climate change has raised concerns about altering the geographic distribution and phenology of ticks that transmit TBDs in Europe and North America (Gray et al., 2009; MacDonald et al., 2020; Ogden et al., 2013; Porretta et al., 2013). Therefore, long-term monitoring is particularly important in areas with cool climates, such as Gifu Prefecture that are bordered area by a SFTS endemic area.

We found a significant association between the dominant tick species, *H. kitaokai*, *H. megaspinosa*, and *H. flava* and PC1 that reflect the abundance of sika deer (Table 2; Fig. 2, Fig. 4). *Cervus nippon* is one of Japanese mammals that host wide range of tick species (Inokuma et al., 2002; Mori et al., 1995; Yamauchi et al., 2009) and all developmental stages of the dominant questing ticks in this study were previously reported infesting on sika deer (Kitaoka et al., 1975; Mori et al., 1990; Yamaguti et al., 1971). Although correlation between deer species density and tick abundance are common in many other countries (Gray et al., 2009; Tsao et al., 2021), abundance of each developmental stage of *H. longicornis* was not always correlated with sika deer population density (Tsukada et al., 2014). Adult ticks are generally found on large mammals (Hoogstraal and Aeschlimann, 1982; Kim et al., 2011; Kitaoka et al., 1975), while immature ticks tend to infest on medium to small mammals such as carnivores and rodents and birds (Ferreira et al., 2023; Oliver Jr, 1989). This may be related to the fact that some species are less likely to be collected by flagging due to their developmental stage, i.e. larval ticks are rarely parasitic on large animals in their immature stage and therefore do not quest on vegetation (Doi et al., 2021; Mysterud et al., 2014; Shibata et al., 2020). Therefore, it would be difficult to estimate tick-bite risk using one single host species given that the risk differs depending on a developmental stage of a species. The role of small mammals, which were not always captured by the camera traps, should also be investigated in future researches by direct trapping of them (Ostfeld et al., 2018) for a better understanding of wildlife contributions to tick abundance. One of the other approach to evaluate the role of mammal host on tick abundance is to use mammal species richness. Some previous studies indicated that higher mammal species richness increase tick abundance (Crandall et al., 2024; Esser et al., 2019). In this study, mammal species richness increased the abundance of adult *H. megaspinosa*, but did not related to it of adult *H. kitaokai*, nymphal *H. flava*, and nymphal *H. megaspinosa* (Table 2). To clarify the inconsistent response of tick abundance to mammal species richness, the relationship among tick abundance evaluated by flagging, tick abundance evaluated by collection from mammal body surface (Doi et al., 2024), and mammal abundance should be examined.

As it mentioned before, *H. longicornis*, *H. flava*, and *A. testudinarium* are suspected vector species of SFTS. The first case of SFTS patient was now reported in Nakatsugawa City, Gifu prefecture in 2025, which locates approximately 50 km to the southeast of Gero City (https://www-pref-gifu-lg-jp.translate.goog/uploaded/attachment/454991.pdf?_x_tr_sl=auto&_x_tr_tl=en&_x_tr_hl=ja&_x_tr_pto=wapp, accessed July 17, 2025). Thus, the result of this study will likely serve as a call to heightened awareness against the SFTS emergence and human tick-bite cases. In addition to the SFTSV vector species, *H*. *flava* and *H*. *longicornis* are also known as vectors of spotted fever group rickettsiae (SFGR) (Fournier et al., 2002; Ishikura et al., 2003; Thu et al., 2019) and *I. persulcatus* and *I. ovatus* are vectors of Lyme borreliosis spirochetes (Kawabata et al., 1987; Miyamoto et al., 1992) in Japan. Although *H. megaspinosa* and *H. kitaoka*i rarely bite humans (Okino et al., 2007, 2008, 2010), they may transfer tick-borne pathogen by co-feeding with other vector tick species via host blood (Randolph et al., 1996). Therefore, although sika deer did not always show a high seroprevalence of SFTSV antibodies, deer population control is likely essential as preventive measure through suppression of tick density for TBDs (Iijima et al., 2022; Matsuyama et al., 2019, 2020; Suzuki et al., 2022; Tabara et al., 2019). However, it should be noted that we did not survey the prevalence of tick-borne pathogens such as SFTSV in ticks and mammals. The effectiveness of population control of mammal species should be evaluated by the integrated survey of tick and mammal abundance and the prevalence of TDBs’ virus in ticks.

## CRediT authorship contribution statement

**Hayato Iijima:** Writing – review & editing, Investigation, Formal analysis, Data curation, Conceptualization. **Kaori Morishima:** Writing – original draft, Investigation. **Hirotaka Komine:** Writing – review & editing, Investigation. **Yuya Watari:** Writing – review & editing, Investigation, Conceptualization. **Kandai Doi:** Writing – review & editing. **Kimiko Okabe:** Writing – review & editing, Supervision, Funding acquisition.

## Declaration of competing interest

The authors declare the following financial interests/personal relationships which may be considered as potential competing interests: Kimiko Okabe reports financial support was provided by ERCA Environmental Research and Technology Development Fund. If there are other authors, they declare that they have no known competing financial interests or personal relationships that could have appeared to influence the work reported in this paper.
