# Supplementary figures and images for: Mammal community composition and season determine the abundance of questing ticks in mountainous forests of central Japan
Source: Int J Parasitol Parasites Wildl. 2025 Jul 21;28:101120. doi: 10.1016/j.ijppaw.2025.101120 (PMC12312105; doi:10.1016/j.ijppaw.2025.101120)

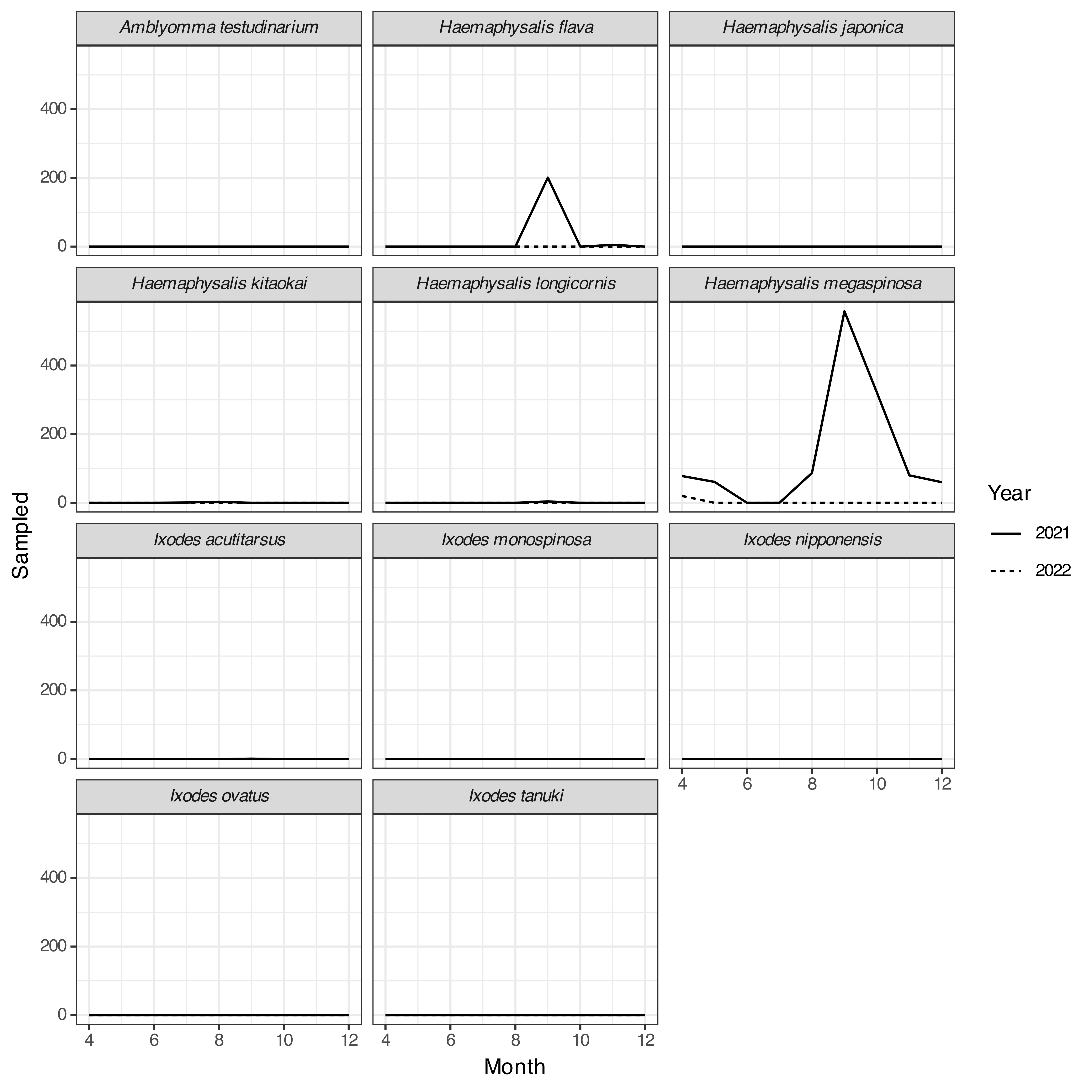


Figure S1 Seasonal difference of sampled larval ticks

Supplement: Multimedia component 1 [file mmc1.docx]
